# Complete mitochondrial genome of *Mustela sibirica* (Carnivora: Mustelidae), a protected and endangered species in China

**DOI:** 10.1080/23802359.2020.1723447

**Published:** 2020-02-07

**Authors:** Wei Gao, Zhihao Lu, Yukang Liang, Zhu-Mei Ren

**Affiliations:** a School of Life Science, Shanxi University, Taiyuan, China;; b College of Plant Protection, Nanjing Agricultural University, Nanjing, China

**Keywords:** *Mustela sibirica*, Mustelidae, mitochondrial genome, phylogeny

## Abstract

We sequenced the complete mitochondrial genome (mitogenome) of *Mustela sibirica* in China by the shotgun genome skimming methods. The mitogenome of *M. sibirica* is 16,558bp in length with the base composition of 32.9% A, 27.3% T, 26.0% C, and 13.9% G, and consists of 13 protein-coding genes (PCGs), 22 tRNAs, two rRNAs, and one non-coding control region. The 13 PCGs use ATG as initiation codons except *ND3*, *ND5* and *ND2* which initiate with codons ATA and ATT, respectively. Four (*COX3*, *ND1*, *ND2* and *ND4*) of the 13 PCGs terminate with a single T– –, and the remainder with a TAA termination codon except *ND3* and *CYT B* using TA– and AGA as termination codon. The phylogenetic tree based on 13 protein-coding genes indicated that *M. sibirica* is sister to the clade grouped with three species *M. nigripes*, *M. eversmannii*, and *M. putorius*, and Mustelinae species formed a monophyletic group, which is close to the Lutrinae clade within Mustelidae.

The mammal species *Mustela sibirica* (Carnivora, Mustelidae, Mustelinae) is a medium-sized weasel native to Asia, where it is widely distributed and inhabits various forest habitats and open areas. The distribution range of *M. sibirica* extends to northern Myanmar, North Korea, Pakistan, Nepal, India, Bhutan, Russia, Taiwan, and northern Thailand (Wilson and Reeder 2005). The species is listed as Least Concern on the IUCN Red List (Abramov et al. 2016). The previous studies on *M. sibirica* mainly focused on its biological characteristics, ecology, and genetic diversity (Suzuki et al. 2013; Son et al. 2017; Zhao et al. 2019). However, not many studies have examined the complete mitogenome of *M. sibirica* in Northern China. Here, we sequenced its complete mitogenome to provide more information on the mitochondrial structure, function, and phylogenetic relationship with other members of Mustelidae.

The muscle material was obtained from a dead adult individual of *M. sibirica*, that was killed by poachers and captured by the forest police in Yangcheng county (112°25′10″E, 35°15′53″N). The specimen (Voucher No. MB-2018M01) and its DNA were stored at the animal herbarium of Manghe National Nature Reserve, Shanxi, China. The genome DNA was sequenced by the shotgun genome-skimming method on an Illumina HiSeq 4000 platform (Zimmer and Wen 2015). The mitogenomic sequence was assembled and annotated within Geneious v11.0.3 using the complete mitogenomes of *M. sibirica* from GenBank as the references. We also performed *de novo* assembly using SPAdes v. 3.7.1 (Bankevich et al. 2012).

The complete mitogenome of *M. sibirica* is a typical circular double-stranded DNA with 16,558bp in length (Accession No. MN206976). The base composition is 32.9% A, 27.3% T, 26.0% C and 13.9% G with the A + T content higher than that of G + C, which is basically consistent with those of other Mustelidae species (Yu et al. 2016). The complete mitogenome of *M. sibirica* totally included 13 protein-coding genes (PCGs, *ND1-ND6*, *ND4L*; *COX1-3*; *ATP6* and *ATP8*; *CYT B*), 22 tRNAs, two rRNAs (12S and 16S rRNA) and one non-coding control region (D-loop), among which *ND6* and six tRNAs are encoded in the reverse-strand, while the remainder in the forward-strand. The 13 PCGs use ATG as initiation codons except for *ND3*, *ND5* and *ND2* initiating with ATA and ATT, respectively. Four PCGs (*COX3*, *ND1*, *ND2* and *ND4*) terminate with a single T– –, and the remainingr with a TAA termination codon except for *ND3* and *CYT B* using TA– and AGA as termination codon, respectively.

We downloaded the complete mitogenomic sequences of Mustelidae species from GenBank with Otariidae species *Arctocephalus townsendi* and *A. pusillus* as outgroups to construct the phylogenetic relationship using RAxML program under the GTR-GAMMA model with 1000 bootstrap replicates (Stamatakis 2014). The results indicated that *M. sibirica* is sister to the clade comprised by the three species *M. oputorius, M. eversmannii*, and *M. nigripes*, and *Mustela* species formed a highly supported monophyletic group close to Lutrinae (Figure 1). The mitochondrial genetic information is very crucial not only to contribute with essential data, but to thoroughly understand the evolutionary history and rare genetic resource conservation of Mustelidae.

**Figure 1.**
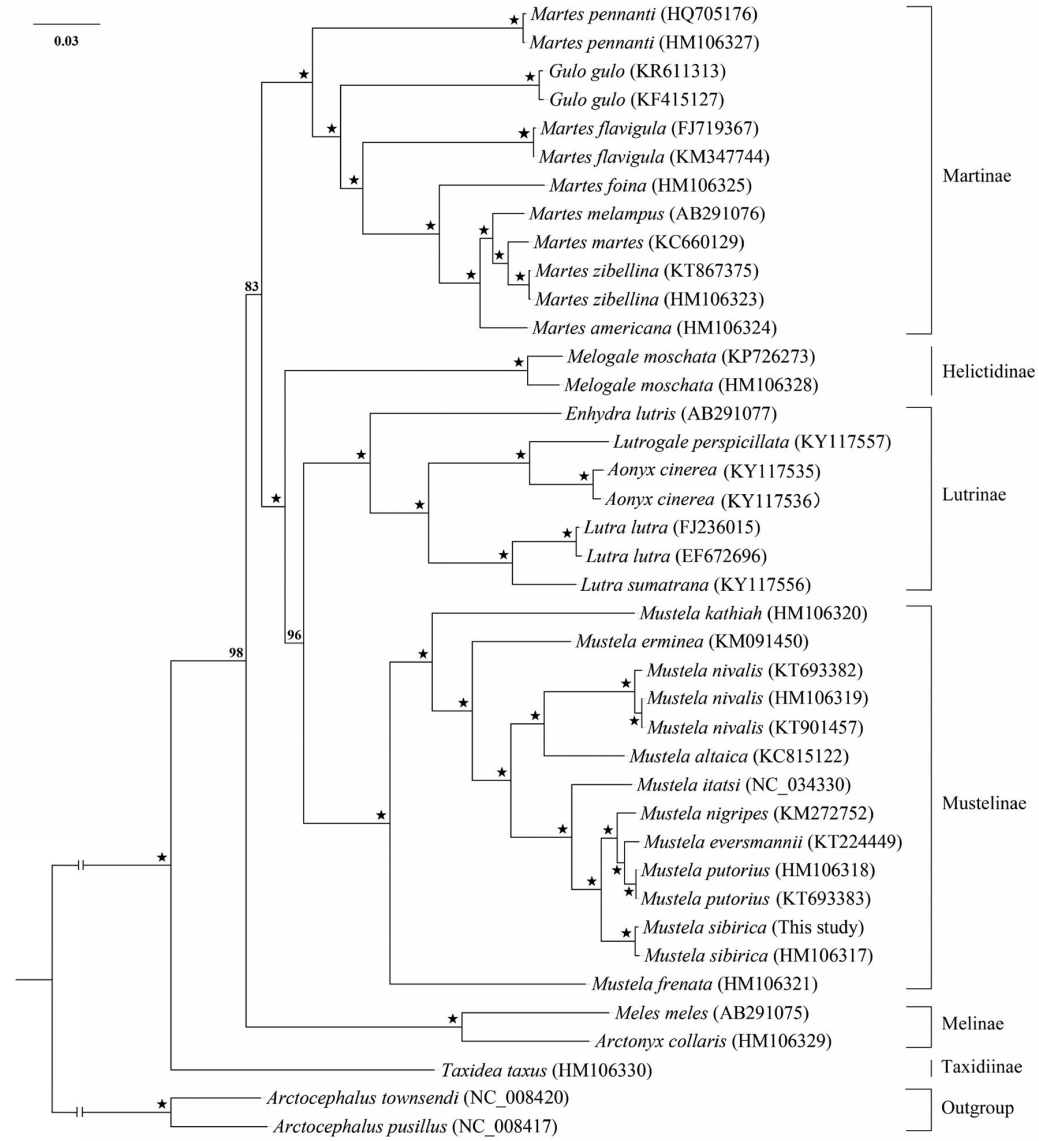
Strict consensus tree of *Mustela sibirica* and other Mustelidae species based on 13 protein-coding genes using RAxML program with *Arctocephalus townsendi* and *A. pusillus* as outgroups. Numbers associated with branches are ML-BS > 70 values, and ‘*’ represents nodes with 100% BS.
